# Individual preferences for the design of a health coaching-based lifestyle promotion program among middle-aged adults in Hong Kong: a discrete choice experiment

**DOI:** 10.1186/s12955-026-02584-y

**Published:** 2026-07-07

**Authors:** Kailu Wang, Cassie Cheuk Ling Lee, Gengcong Qu, Cyrus Tak Ka Tang, Hei Hang Edmund Yiu, Xue Yang, Carmen Ka Man Wong, Samuel Yeung-shan Wong, Benjamin Hon Kei Yip

**Affiliations:** 1 Jockey Club School of Public Health and Primary Care, Faculty of Medicine, The Chinese University of Hong Kong, Hong Kong, Hong Kong SAR China; 2 Centre for Safe Medication Practice and Research, Department of Pharmacology and Pharmacy, LKS Faculty of Medicine, The University of Hong Kong, Hong Kong, Hong Kong SAR China

**Keywords:** Discrete choice experiment, Health coaching, Lifestyle modification, Public preferences

## Abstract

**Objectives:**

Health coaching is an effective approach to motivating lifestyle modifications and preventing the onset of non-communicable diseases. This study aims to examine middle-aged adults’ preferences for health coaching programs to improve public acceptance of these programs.

**Methods:**

A discrete choice experiment (DCE) was conducted among adults aged 35–59 years in Hong Kong who were recruited from a population-based cohort established to evaluate outcomes related to long COVID. The DCE attributes and levels were selected based on a literature review and qualitative interviews (*n* = 10) with users and providers from a local health coaching program. The selected attributes included program duration, delivery mode of coaching sessions, frequency of coaching sessions, availability of blood tests, core program format, smartwatch usage, and out-of-pocket payment. An online survey with 10 choice sets was conducted to collect participants’ choices regarding health coaching services with (copayment scenario, 10 choices) and without out-of-pocket payment (no copayment scenario, 10 choices). A mixed logit model and a latent class model were used for the analysis.

**Results:**

A total of 912 responses were collected, of which 554 valid records were included in the analysis. In the copayment scenario, participants preferred programs that provided a smartwatch and a lower copayment rate. In the no copayment scenario, participants preferred a moderate coaching frequency, communication via phone calls or text messages, and provision of a smartwatch. Distinct preference groups were identified, including those who preferred face-to-face versus remote modes of delivery, high versus low contact frequency, and those who were sensitive versus insensitive to cost.

**Conclusions:**

Lower copayment and smartwatch provision improve acceptance of health coaching programs. Substantial preference heterogeneity for program duration, health coaching frequency, and delivery mode was found. While material incentives are important for improving acceptance of health coaching, considerable preference heterogeneity for non-monetary attributes suggests that options for health coaching program intensity can be offered along with subsidies or lower out-of-pocket payment to promote these services.

**Supplementary Information:**

The online version contains supplementary material available at https://doi.org/10.1186/s12955-026-02584-y.

## Introduction

Non-communicable diseases (NCDs) were responsible for an estimated 73% of deaths worldwide in 2021 [1]. Unhealthy lifestyles, including alcohol consumption, physical inactivity, smoking, and an unhealthy diet, are considered one of the major drivers of NCD development [2]. Recent evidence also suggests a shift toward earlier NCD onset, with an increasing risk of NCD emerging during middle age (35–59 years) [3, 4]. Middle age is therefore a critical period for establishing and maintaining healthy habits. Studies indicate that interventions during middle age can substantially delay or prevent NCD onset, which often occurs after age 60 [5, 6].

Implementing and maintaining lifestyle changes depends largely on individual psychological factors. Pre-intentional (motivational) factors, such as beliefs, attitudes, risk perceptions, outcome expectancies, motivation, and perceived barriers, strongly influence whether an individual intends to change [7]. Post-intentional (volitional) factors, such as self-efficacy, action and coping planning, and self-regulatory skills, affect whether behaviour change is initiated and sustained over time [7]. Health coaching, which is an essential part of lifestyle medicine, is an individualised, person-centred approach to conducting health education and health that identifies individual strengths and motivations in lifestyle behaviours [8, 9]. Existing literature suggests that health coaching can enhance individual physical activity levels and weight management, increase self-efficacy for achieving health-related goals, and improve physical health, mental health, and quality of life [8, 10].

To date, substantial research and implementation efforts have focused on developing lifestyle modification programs, including health coaching interventions. One scoping review identified studies published between 2007 and 2017 that focused on promoting healthy behaviour and weight management, primarily among older adults and other vulnerable groups (e.g., individuals with obesity or chronic diseases). These interventions were commonly informed by psychological frameworks such as motivational interviewing (MI) and social cognitive theory, and varied widely in delivery mode, duration, and format (individual- vs. group-based) [11]. In the local context of Hong Kong, a four-arm randomised controlled trial conducted in 2024 integrated health coaching with a health wearable, recruited 1,000 middle-aged adults at risk of developing chronic diseases, and followed participants for six months [12].

To improve the person-centredness of health coaching programs and enable them to meet the public’s needs and expectations, it is important for service providers to understand how to design and deliver the program. Appropriate program design could also increase public acceptance and adherence to the program, thereby achieving wider coverage of the intervention. One useful way to assess these preferences is the discrete choice experiment (DCE), which can quantify the relative importance of different program attributes. However, preferences for intervention design and delivery characteristics have seldom been explored in earlier DCEs on related topics across different populations, including the Hong Kong population. A recent systematic review summarised DCE studies investigating adults’ preferences for, or acceptability of, lifestyle modification interventions. However, most included studies focused on individuals with specific chronic diseases or behavioural risk factors, such as type 2 diabetes mellitus (T2DM), smoking, or physical inactivity [13]. Only three studies in the review examined preferences among the general adult population [14–16]. In addition, one recent DCE evaluated preferences for the delivery mode and key features of a weight-loss program among adults with T2DM or at risk of T2DM [17]. Earlier DCEs also considered attributes such as cost and financial incentives in lifestyle modification programs, although these studies also focused on patients with T2DM [18, 19]. Overall, the current literature suggests that there remains a gap in the DCE literature regarding the general public’s perceptions of, and preferences for, prevention-oriented lifestyle intervention programs.

In this study, we aimed to elicit the preferences of middle-aged adults in Hong Kong for a health coaching-based lifestyle modification program and examine preference heterogeneity across individuals. Such information would be useful for designing a person-centred primary prevention program that is acceptable to the public and for incorporating the preferences of middle-aged adults into a subsequent intensive, iterative participatory procedure with stakeholders to develop and refine the health coaching program.

## Methods

This was a cross-sectional online survey that incorporated a DCE conducted among middle-aged adults in Hong Kong. The study was approved by the Survey and Health Behavioural Research Committee of the Chinese University of Hong Kong (Reference number: SBRE-24–0374 A). The results of this study were reported in accordance with the DIRECT checklist for reporting DCEs [20].

### DCE design

A DCE is a survey-based instrument used to quantify preferences for a series of attributes of a good or service by asking participants to choose among several hypothetical alternatives described by different combinations of attribute levels. The attributes and their levels were determined following a structured procedure. First, a literature review was conducted. Identified systematic reviews showed that interventions combining health coaching interventions on lifestyle factors and digital health solutions led to promising behavioural and health improvements [21–23]. Second, a descriptive framework was established based on the practice of a recent large-scale community-based health promotion program in the local context, i.e., The Jockey Club “We Watch” Healthy Lifestyle project [12]. This was an evidence-based, protocol-driven project comprising four major components, including health coaching, blood tests, core program, and use of a smartwatch. A series of semi-structured interviews was conducted with participants (*n* = 5), coaches (*n* = 3), and administrators (*n* = 2) of this project. The interviewees were asked to indicate their perceived importance of the features in the four major components. Their perception of the design and delivery of these components was collected. Important features of the project, as indicated, included preferred duration, frequency (intensity), format of coaching, blood tests, learning mode (passive or interactive), core program format (face-to-face or remote), smartwatch usage, and provision of the smartwatch. Features that were not deemed important included flexibility of coaching, risk assessment questionnaire, other factors of the core program, and data sharing.

The interview findings were then discussed by a group of researchers and health coaches for attribute selection and refinement. Seven features were selected as the DCE attributes, including the program duration, mode of delivery for coaching sessions, frequency of coaching sessions, availability of blood tests, core program format, smartwatch availability, and out-of-pocket payment for the whole program. The attributes and levels were listed in Table 1 according to a DCE reporting guideline [24]. Out-of-pocket payment was computed as the product of the costs of the other attribute levels and the copayment rate [25]. To be specific, the overall costs of the program were calculated based on the attribute levels of that specific alternative (adding up the costs of the four components: coaching, blood tests, core program, and smartwatch) and the associated unit cost. The unit costs were determined based on real-world data by the same group of researchers and health coaches for attribute selection. The presented out-of-pocket payment was calculated by multiplying the total cost by the attribute copayment rate presented in the right-hand column of Table 1. Using this method, the copayment amount varied even if the other attribute levels remained the same. Thus, the calculated amount was closer to the real-world price, which enabled the capture of a more realistic choice behaviour.

**Table 1:** Attributes and levels of the discrete choice experiment

Attributes	Abbreviation	Levels
Program duration	Duration	3 months
		6 months
		12 months
Mode of delivery for coaching sessions	Coachingformat	Face-to-face
		Videoconferencing except the first and last session
		Via WhatsApp or phone call except the first and last session
Frequency of coaching sessions	Coachingfreq	Every 2 weeks
		Every 3 weeks
		Every 4 weeks
Availability of a blood test	Bloodtest	No such test
		One blood test at baseline
		Two blood tests at baseline and upon completion of the program
Core program format	Coreprogram	Face-to-face
		Videoconferencing except the first and last session
		Videos for the lectures (not interactive)
Use of smartwatch	Watch	Watch not necessary for the program
		Use one’s own watch
		Watch provided by the program
Out-of-pocket payment (the product of cost of the corresponding alternative and one of the three levels of copayment rate on the right)	Payment	50%
		75%
		100%

As it is not practically feasible to include all combinations of the attribute levels (k = 2,390,391) in the survey, a D-efficient design without prior means was adopted to generate the choice sets (Stata package: dcreate; D-efficiency score: 5.749). A total of forty choice sets were generated, with two health coaching alternatives in each choice set. The forty choice sets were further assigned to four blocks, with ten choice sets in each block, to alleviate the cognitive burden of the survey participants. According to Johnson and Orme’s rule of thumb for sample size estimation, this design required at least 75 valid responses [26]. In each task, participants were asked to choose their preferred alternative twice: first, with the copayment amounts shown (“copayment” scenario), then, assuming no copayment (i.e., ignoring the cost attribute; “no copayment” scenario). The latter was used to elicit preferences for non-monetary attributes and to reduce the potential dominance of the monetary attribute. An opt-out option was included for each choice set, allowing participants to indicate that they preferred neither of the two alternatives and decided not to join the health coaching program. The order of the choice sets was randomised across individuals to minimise the sequence effects. Background information and hypothetical scenarios were presented before the DCE tasks to provide context for the participants’ choices. Participants were familiarised with the tasks and attribute levels with a warm-up DCE task presented before the formal tasks.

Pre-testing of the questionnaire was conducted with five participants who met the inclusion criteria to refine the wording and format of the questionnaire. Based on the feedback, we shortened the introduction and highlighted the key parts in bold text for easier reading. Instructions were clarified on each page of the DCE choice sets. A progress bar was added. Respondents’ feedback on the improved questionnaire was manageable. An example of a choice set was presented in Table 2.

**Table 2:** An example of the discrete choice set

	Health project 1	Health project 2	Neither one
Program duration	12 months	3 months	I would join NEITHER of them
Mode of delivery for coaching sessions	Face-to-face	Via WhatsApp or phone call except the first and last session	
Frequency of coaching session	Every 4 weeks	Every 2 weeks	
Availability of a blood test	One blood test at baseline	No such test	
Core program format	Face-to-face	Videos for the lectures (not interactive)	
Use of smart watch	Watch not necessary for the program	Use one’s own watch	
Out-of-pocket payment (whole program)	HKD 1890	HKD 882	
a) Which of the two health projects would you prefer? (1) Health project 1, (2) Health project 2, (3) Neither one			
b) Assuming both projects are free of charge (i.e., out-of-pocket payment is HK$0), Which of the two health projects would you prefer? (1) Health project 1, (2) Health project 2, (3) Neither one			

### Participants and data collection

The survey targeted Hong Kong residents aged 35–59 years, including those with and without chronic conditions. Individuals who could not provide informed consent were excluded. The recruitment primarily utilised a panel of potential participants, which was maintained by our research team and included adults from a population-based cohort designed to capture outcomes of long COVID. Participants who had previously consented to being approached for future research were invited via a survey link sent through a messaging platform, ensuring easy access to the survey. Participants were asked to complete a self-administered online survey through the invitation link after providing consent to participate in the study. A research staff member was available through the messaging platform for the participants to ask questions about the survey. Participants who completed the survey received supermarket coupons worth HK$40 as incentives.

### Questionnaire

In addition to the DCE tasks, the questionnaire also included the following items: (1) socio-demographic characteristics, (2) occupations and work-related factors, including working hours, long sitting hours, and night shifts, (3) health status, including height, weight, hypertension, hyperglycemia, hypercholesterolemia, and chronic disease diagnoses, and (4) lifestyle behaviours, including smoking, alcohol consumption, diet, and physical activity.

### Statistical analysis

The distribution of sample characteristics was summarised. The random utility theory formed the basis of DCE analysis, which assumed the utility (U) of choosing an alternative could be described by a deterministic component (V) and a random component (ε) [27]. The utility of individual i for choosing a health coaching alternative could be specified as

$$\:{U}_{i}={V}_{i}+\:{\epsilon\:}_{i}$$

$$\:{V}_{i|opt-in}=\:{\beta\:}_{i0}+{\sum\:}_{k=1}^{K}{{\beta\:}_{ik}A}_{k\:}$$

$$\:{V}_{i|opt-out\:}=0$$

Where the $\:{A}_{k\:}$referred to the vector of attribute levels, $\:{\beta\:}_{ik}$ referred to the vector of model coefficients for individual I, and $\:{\beta\:}_{i0}$ referred to the alternative-specific constant for the opt-out option. The dichotomous choice on each alternative was used as the dependent variable of the regression, with utility treated as a latent construct. Mixed logit (MIXL) models were first applied to model the DCE choices under the copayment and no-copayment scenarios separately. In MIXL models, all model coefficients, β, were assumed to account for preference heterogeneity. Effect coding was used to code the attribute levels (an example was provided in Supplementary Table A1), and the copayment amount was included as a continuous variable. With effect coding, the model coefficients were not correlated with the intercept of the model, avoiding misinterpretation of the intercept [28]. It also avoided arbitrarily setting a reference level [28]. A positive coefficient indicated the respondents preferred the attribute level compared with the average preference across levels of that attribute, while a negative coefficient indicated a lower preference. The relative importance of each attribute was estimated by calculating the ratio of the range of its MIXL model coefficients to the sum of the coefficient ranges across all attributes [29]. Willingness to pay (WTP) was estimated for attribute levels with statistically significant coefficients by dividing the coefficient for the attribute level of interest by the coefficient for out-of-pocket payment. After the MIXL model was estimated, a latent class logit (LCL) model was used to identify respondent segments with heterogeneous preferences [30]. The number of classes was determined using the Bayesian information criterion (BIC) of the models [31]. Age, sex, education, and occupation were added to the LCL models as class membership factors to show the association of different segments with these factors, as they are basic socio-demographic factors that can be used by program providers in identifying target audiences and tailoring the services to them. To further assess the relationship between class membership and various factors, posterior probabilities of assignment to each class were estimated based on the LCL model, and their associations with lifestyle and health factors were examined using chi-square tests. To exclude potential non-engagement, participants who consistently chose the same alternative (alternative 1 or 2) in all 20 choice tasks (10 choices in the copayment scenarios and 10 in the non-copayment scenarios) or consistently chose the opt-out in all 10 non-copayment scenarios were excluded from analysis (consistently choosing of opt-out in all 10 copayment scenarios were deemed possible as some individuals might not be willing to pay for a health coaching program). As the choices in the non-copayment scenarios followed the those in the copayment scenarios and respondents were exposed to the copayment information, it was necessary to confirm whether the copayment information affected non-copayment choices. *P* < 0.05 was considered statistically significant. All statistical analyses were conducted using Stata 18.0.

Several sensitivity analyses were conducted. First, we examined the impact of copayment amount (as an independent variable) on choices in the “no-copayment” scenario (dependent variable) in a MIXL model with the other attribute levels as the independent variables, because copayments were assumed not to influence choices in this scenario (i.e., the coefficient should be close to zero). Second, for the attribute of out-of-pocket payment, the copayment rate (50%, 75%, and 100%) was included in the model to replace the copayment amount as the independent variable. The respondents were presented with a copayment amount calculated by multiplying the overall cost (determined by other attribute levels) by the copayment rate, which factored into respondents’ choices. This sensitivity analysis was intended to capture the effects of cost-related attribute levels, as the presented cost amount was not controlled in the model. By contrast, the primary analysis using the copayment amount as an independent variable estimated the preferences for attribute levels after controlling for costs, which captured the preferences for non-monetary components of the attribute levels that were isolated from cost considerations. Third, to account for non-participation, two-class LCL models were estimated for the copayment and non-copayment scenarios separately, with the coefficients for the attribute levels constrained to be zero in one of the classes (i.e., assuming that non-engaged participants tended to choose randomly and did not present any tendency on the attribute levels). In these models, all preference weights were constrained to zero in one of the two classes referred to those who were not actively participating in the choice tasks, and the other class represented the preference weights of the remaining sample after segregation of non-participation responses. Fourth, mixed logit models were estimated after further excluding respondents who consistently chose alternatives in the same position (either all left-hand side or right-hand side alternatives) in either scenario, to account for potential non-participation in the DCE survey.

## Results

### Sample characteristics

A total of 1,015 invitations were sent to individuals within the eligible age range. After removing duplicates, 912 responses remained. Of these, 61 were excluded because the quota limits for their age–sex groups had been reached, and the remaining 851 were eligible and agreed to participate in the survey. Overall, the response rate was 83.8%. Among the eligible participants, 656 completed the DCE tasks. After excluding those who consistently chose the same alternative in all choice sets (*n* = 102), 554 valid responses were included in the analysis. Of them, 372 (67.2%) were female, and 231 (41.7%) were aged 45–59 years. More than half (57.8%) had attained a bachelor’s degree or higher, and 69.0% worked full-time (Table 3). The sample included a higher proportion of females and a higher proportion of individuals with higher educational levels than the general middle-aged population in Hong Kong.

**Table 3:** Sample characteristics

	*N*	Percentage (%)
**Age**		
35–44 years	323	58.3
45–59 years	231	41.7
**Sex**		
Female	372	67.2
Male	182	32.9
**Education**		
Secondary school or below	226	42.2
Higher education	309	57.8
(Missing)	19	
**Employment**		
Other than full time job	172	31.1
Full time job	382	69.0
**Monthly household income**		
Below HK$40,000	275	54.4
HK$40,000 or above	231	45.7
(Missing)	48	
**BMI**		
< 18.5	59	10.7
18.5–22.9	257	46.6
23-24.9	78	14.2
>=25	157	28.5
(Missing)	3	
**Diagnosis of high cholesterol**		
No	447	80.7
Yes	107	19.3
**Diagnosis of hypertension**		
No	474	85.6
Yes	80	14.4
**Diagnosis of diabetes**		
No	513	92.6
Yes	41	7.4
**Time of moderate-intensity physical activity per week**		
Lower than 150 min	482	87.0
150 min or above	72	13.0
**Intake of vegetables or fruits > = 4.5 cup**		
No	186	33.6
Yes	368	66.4
**Regular use of a smart watch**		
No	324	58.5
Yes	230	41.5
**Total**	554	100.0

### Copayment scenario

Table 4 presents mixed logit (MIXL) estimates (means and standard errors) and preference heterogeneity (standard deviations) for the health coaching attributes under the copayment and no-copayment scenarios. On average, the use of a smartwatch and a change in the out-of-pocket payment amount significantly affected respondents’ choices. Respondents preferred having the smartwatch provided by the program and paying a lower out-of-pocket amount. Using the MIXL coefficients, each HK$1,000 increase in out-of-pocket payment was estimated to reduce the probability of acceptance by around 14% points on average. Respondents were willing to pay HK$563 (95% CI: HK$431–696; US$1 = HK$7.8) for the smartwatch provided by the program, and willing to pay HK$490 (95% CI: HK$347–632) to avoid a program that required them to use their own smartwatch. These two attributes were also relatively more important than the other attributes (Supplementary Figure A1). The preference weights of the other attribute levels were not statistically significant, while substantial preference heterogeneity was observed for program duration, format of coaching, availability of blood test, format of core program, and out-of-pocket payment level.

**Table 4:** The mixed logit model results in both scenarios

	“Copayment” scenario		“No copayment” scenario	
	Mean (SE)	SD (SE)	Mean (SE)	SD (SE)
Duration: 3 months	0.08 (0.05)	0.42 (0.06) *	-0.03 (0.04)	-0.07 (0.03) *
Duration: 6 months	-0.02 (0.04)	0.02 (0.07)	0.02 (0.03)	0.06 (0.06)
Duration: 12 months	-0.05 (0.05)	0.42 (0.06) *	0.01 (0.04)	0.51 (0.04) *
Coachingformat: Face-to-face	0.02 (0.05)	0.57 (0.06) *	-0.10 (0.04)	0.50 (0.04) *
Coachingformat: Videoconference	-0.04 (0.04)	0.25 (0.07) *	0.00 (0.03)	-0.18 (0.05) *
Coachingformat: WhatsApp/call	0.02 (0.04)	0.51 (0.06) *	0.10 (0.03) *	0.47 (0.04) *
Coachingfreq: every 2 weeks	-0.02 (0.04)	0.05 (0.06)	-0.07 (0.03) *	0.50 (0.04) *
Coachingfreq: every 3 weeks	0.01 (0.03)	-0.05 (0.06)	0.06 (0.03) *	-0.04 (0.05)
Coachingfreq: every 4 weeks	0.00 (0.04)	0.02 (0.05)	0.01 (0.03)	0.07 (0.05)
Blood test: No such test	-0.03 (0.04)	0.16 (0.07) *	-0.03 (0.03)	0.50 (0.04) *
Blood test: One test	0.01 (0.04)	-0.12 (0.08) *	0.02 (0.03)	-0.13 (0.07)
Blood test: Two tests	0.02 (0.04)	-0.12 (0.06)	0.02 (0.03)	-0.15 (0.07) *
Coreprogram: Face-to-face	0.08 (0.04)	0.30 (0.05) *	-0.03 (0.03)	0.50 (0.04) *
Coreprogram: Videoconference	-0.07 (0.04)	0.27 (0.05) *	0.02 (0.03)	0.26 (0.04) *
Coreprogram: Videos	-0.01 (0.04)	0.13 (0.05) *	0.01 (0.03)	0.14 (0.07)
Watch: Not necessary	-0.04 (0.04)	0.03 (0.06)	-0.07 (0.03) *	0.50 (0.04)
Watch: One’s own watch	-0.28 (0.04)*	-0.01 (0.07)	-0.41 (0.03) *	-0.01 (0.06)
Watch: Provided by program	0.32 (0.04) *	0.03 (0.06)	0.48 (0.03) *	-0.36 (0.04)
Out-of-pocket payment (every $100)	-0.06 (0.01) *	0.04 (0.00) *	**-**	-
Opt-out	-1.56 (0.23) *	6.13 (0.35) *	-3.41 (0.23) *	2.66 (0.19)

Note: SD: Standard deviation; SE: Standard error; * Represents significant result with *P* < 0.05

**Fig. 1:**
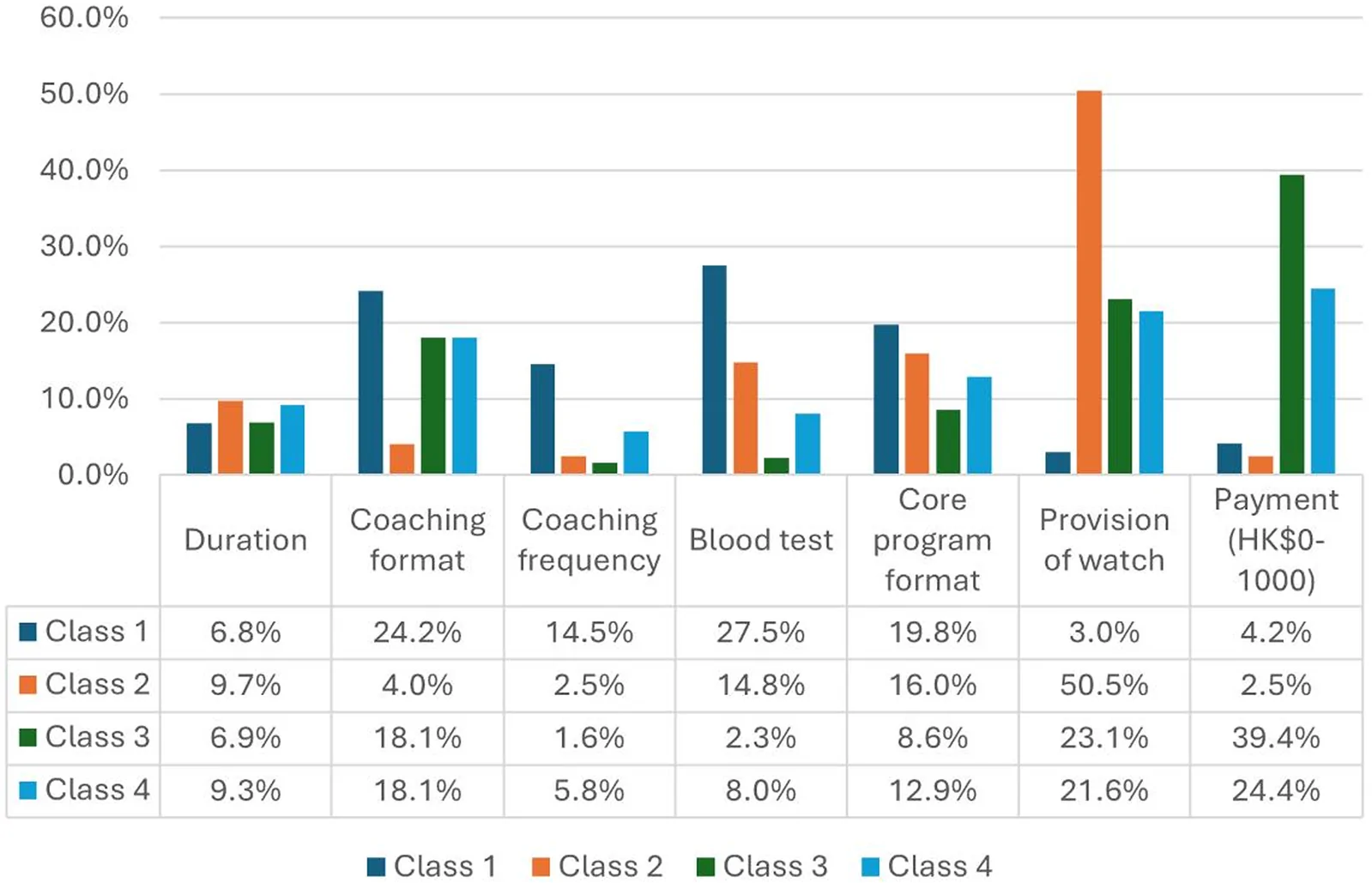
“Copayment” scenario: the relative importance of attributes across different latent classes. Note: the percentages refer to the relative importance of the attributes in respective latent classes. Preference pattern: Class 1: high refusal; Class 2: smartwatch-focused; Class 3: cost-sensitive and remote-mode-oriented; Class 4: face-to-face oriented

The LCL model identified four distinct classes of individuals with different preference patterns for health coaching (Fig. 1 and Supplementary Table A2). In Class 1 (class share: 36.3%), respondents showed a high likelihood of opting out of the health coaching programs. Even when they did not opt out in some choice sets, they preferred to minimise interactions, including alternatives with no blood tests and those offering the core program via videos. In contrast, participants in Class 2 (class share: 26.3%) tended to join health coaching programs and appeared to be cost-insensitive, and focused on whether the smartwatch was provided by the program. In Class 3 (class share: 21.9%), participants were relatively cost-sensitive and prioritised having a smartwatch provided by the program, and also preferred to receive health coaching through messaging or phone calls. In Class 4 (class share: 15.5%), participants preferred face-to-face interactions in health coaching sessions and the core program. Compared with Class 1 (high refusal), Classes 2, 3, and 4 had higher proportions of females. There were no significant differences among the classes in age group, education level, or employment status. Based on posterior class-membership probabilities, individuals in Class 1 (high refusal) were more likely to be of normal weight or underweight. Those in Class 2 (smartwatch-focused) and Class 4 (face-to-face oriented) were more likely to have a healthy diet (i.e., consuming sufficient vegetables and fruits) than those in the other classes. Those in Class 3 (cost-sensitive and remote-mode-oriented) tended to use a smartwatch regularly.

### No copayment scenario

The health coaching preferences were examined under the no-copayment scenario. In the MIXL model results (Table 4), the participants prioritised the provision of a smartwatch, followed by health coaching format and health coaching frequency. Providing health coaching through messaging or calls, and delivered every 3 weeks, was more preferable. No statistically significant preferences were observed for program duration, availability of blood tests, or format of core programs, despite substantial preference heterogeneity being observed for these attributes.

**Fig. 2:**
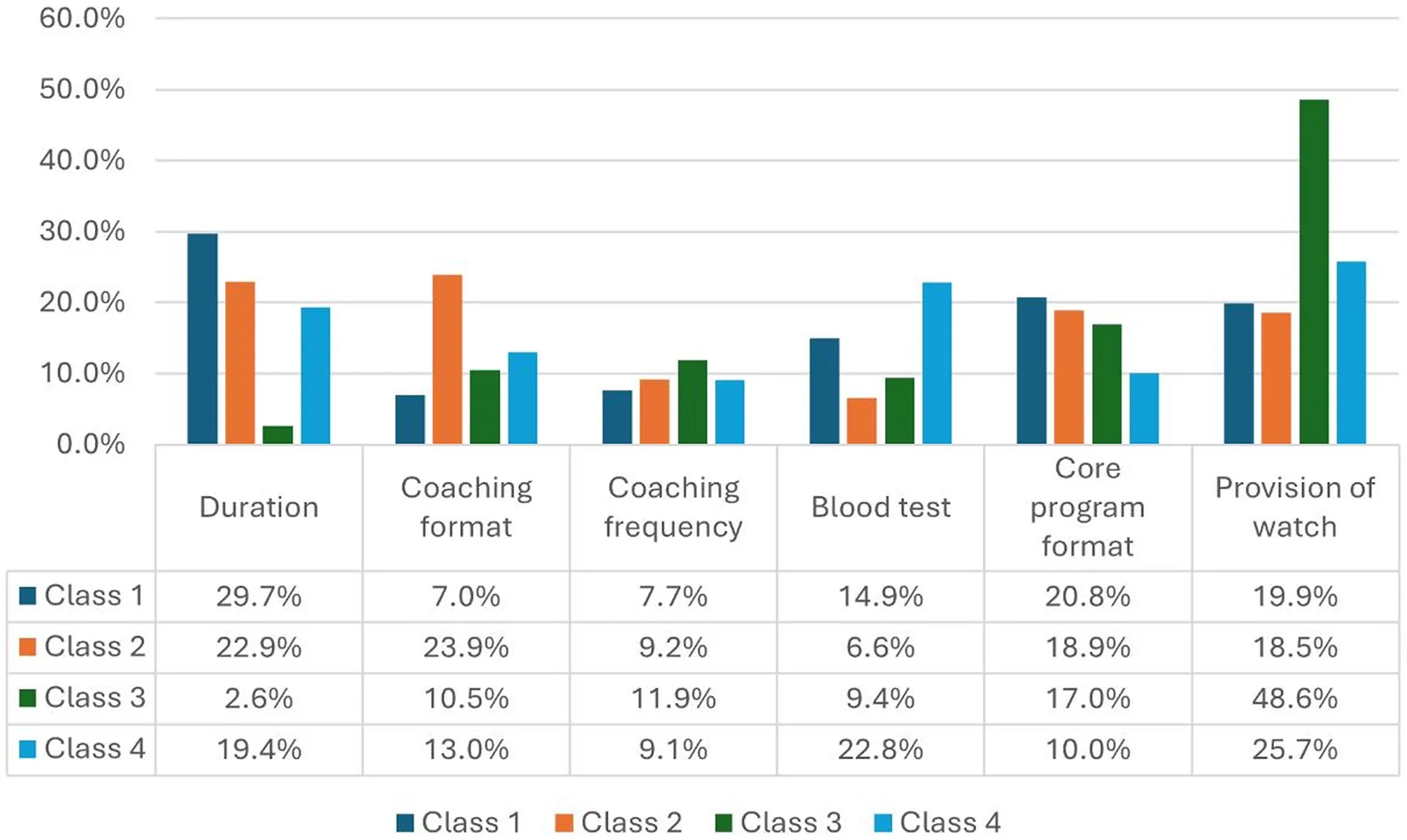
“No copayment” scenario: the relative importance of attributes across different latent classes. Note: the percentages refer to the relative importance of the attributes in respective latent classes. Preference pattern: Class 1: high coaching intensity; Class 2: low coaching intensity; Class 3: smartwatch-focused; Class 4: no-blood-test-preferred

The LCL model also identified four classes of individuals with heterogeneous preferences (Fig. 2 and Supplementary Table A3). In Class 1 (class share: 38.6%), participants preferred having more intensive health coaching, as reflected by longer program duration, more frequent coaching, and face-to-face participation in the core program. Meanwhile, Class 2 (class share: 34.2%) participants preferred less intensive health coaching, including shorter program duration, lower coaching frequency, and avoiding face-to-face participation in both health coaching and core program. In Class 3 (class share: 15.5%), participants focused on having a smartwatch provided by the program, which was substantially more important than the other attributes. Class 4 (class share: 11.7%) participants tended to prefer shorter program duration and no blood tests, while preferring to receive a smartwatch from the program. The main difference between Class 4 (no-blood-test-preferred) and Class 2 (low coaching intensity) was that the latter showed less preference for the availability of blood tests and preferred not to receive a smartwatch from the program. Compared with Class 4 (no-blood-test-preferred), Class 3 (smartwatch-focused) had a higher proportion of females, while Class 1 (high coaching intensity) had a higher proportion of participants aged 45 years or above. Moreover, those in Class 3 (smartwatch-focused) were less likely to have sufficient vegetables and fruits.

### Sensitivity analysis results

The first sensitivity analysis showed that the copayment amount presented to respondents did not affect respondents’ choices in the no-copayment scenario (Supplementary Table A4), which aligned with the assumption. The second sensitivity analysis showed respondents’ preference for attribute levels after accounting for their associated prices (as the cost amount was presented in the choice sets but not adjusted in the model), whereas the primary analysis showed respondents’ preferences for the non-monetary attributes that were independent from the prices.

The sensitivity analysis that replaced copayment amount with copayment rate as an independent variable showed that respondents preferred shorter program duration, coaching delivered through messaging or calls, lower coaching frequency, no blood tests, no videoconferencing for the core program, and having a smartwatch provided by the program, after accounting for the lower costs associated with most of these attribute levels. In contrast, the original primary analysis suggested that respondents did not have a preference for most of these attribute levels, except for having a smartwatch provided by the program, after controlling for the associated costs (Supplementary Table A5). The most important attributes identified in the sensitivity analysis were program duration, followed by smartwatch, coaching frequency, coaching delivery mode, availability of blood tests, and core program format. Compared with the primary analysis, program duration and coaching frequency had greater relative importance in the sensitivity analysis. Longer program duration and higher coaching frequency were associated with much greater costs; therefore, the respondents were inclined to choose a shorter program to reduce costs.

The third sensitivity analysis, which used two-class LCL models to segregate respondents with potential non-participation, showed that more attribute levels, including coaching format and core program format in the copayment scenario, as well as program duration and availability of blood tests in the no-copayment scenario, became statistically significant (Supplementary Table A6). It suggested that non-participation by respondents could be an important factor influencing the model outcomes. Respondents with less random choice patterns across the DCE alternatives (i.e., those in the second class) were found to prefer face-to-face coaching and to avoid videoconferencing in coaching sessions or the core program in the copayment scenario, compared with the absence of significant preferences for these attributes in the primary analysis. This pattern may reflect that the respondents in the second class, who might have better mental capacity due to more consistent responses for the DCE tasks, potentially have stronger preferences for active, in-person participation in the coaching sessions and core program. The fourth sensitivity analysis of MIXL that excluded additional respondents with potential non-participation showed very similar results compared with the primary analysis (Supplementary Table A7).

## Discussion

This analysis of the DCE data among middle-aged adults in Hong Kong revealed preferences for a health coaching program for lifestyle promotion. On average, program acceptance was significantly influenced by whether a smartwatch was provided and the required out-of-pocket payment amount. The delivery mode and frequency of health coaching also influenced acceptance when copayment was not included in the model. Moreover, four groups of respondents with distinct preferences for smartwatch provision and the extent of program participation were identified in both the copayment and no-copayment scenarios. Among the identified preference groups, high-refusal individuals in the copayment scenario tended to be male. In the non-copayment scenario, a higher proportion of women focused on smartwatch provision, and a higher proportion of participants aged 45–59 years preferred a high intensity of participation.

In both the copayment and no-copayment scenarios, provision of a smartwatch was consistently identified as an influential attribute associated with a greater likelihood of accepting health coaching among the middle-aged. One possible explanation is that respondents perceived the smartwatch as a participation incentive or reward. In a previous DCE study, the public considered provision of incentives such as subsidies for vegetables and fruits to be the most important policy option for promoting healthy diets [32]. Offering financial rewards to patients with diabetes has been shown to facilitate participation in a lifestyle modification program [33]. By contrast, the use of one’s own smartwatch was less preferred. Despite the smartwatch being considered useful in motivating users to engage in healthy behaviour and providing users and healthcare professionals with health data that is difficult to obtain [34], our findings suggest that respondents were reluctant to make extra financial investments for purchasing a smartwatch.

The amount of out-of-pocket payment strongly influenced respondents’ intention to join the program. The relative importance of out-of-pocket payments for respondents’ acceptance was similar to that of the smartwatch attribute when the payment amount changed from HK$0 to HK$1,000 for the entire program. The provision of financial incentives was considered a promising strategy for improving participation in health promotion activities. A meta-analysis reported that the use of incentive strategies was associated with a higher enrolment and retention rate of various health promotion programs, and these strategies were more effective when cash incentives were combined with non-monetary rewards [35]. Nevertheless, although financial incentives may help increase participation in the programs, it remains uncertain whether they ultimately lead to sustained changes in health behaviours. Their effectiveness may depend on several factors, including outcome measures used for evaluation, incentive structure, overall program design, and the type of reward offered [36]. In addition, financial incentives may be more effective when designed according to principles from behavioural economics, such as loss aversion and hyperbolic discounting [37]. Similarly, providing a smartwatch may not be sufficient to induce behavioural changes; the design of the program and the strategies for engaging participants in healthy behaviours also play an important role [38].

Other program attributes, despite being less important than smartwatch provision and copayment, should also be considered in relation to individuals’ preferences. First, while the primary analysis suggested that overall, respondents did not show statistically significant preferences for program duration, coaching delivery mode, coaching frequency, blood test availability, or core program format after controlling for copayment amount, the second sensitivity analysis showed respondents preferred lower-cost attribute levels when copayment amount was taken into account. That included shorter program duration, text message or phone-based coaching, lower coaching intensity, and the absence of a blood test. These findings suggest that although these program attributes may be less important to the middle-aged in scenarios where the user copayment is not directly linked to service costs (e.g., a universal amount is applied, or the copayment is determined by user’s ability to pay rather than service costs), increasing costs and copayment associated with specific program attributes under a cost-based pricing scenario (the copayment amount is determined by or correlated with the costs of the program) may reduce the public acceptance of the program. Second, although the average preferences for most of these attribute levels were not statistically significant, distinct preference patterns were observed across individuals. In the copayment scenario, apart from respondents who were inclined to refuse the program and nearly half of the respondents who focused mainly on smartwatch provision and copayments, a small group of respondents preferred face-to-face delivery for both coaching and the core program. This finding is consistent with a DCE study on non-pharmacological diabetic care, in which patients preferred individual in-person education to digital health-based education [39]. Moreover, in the co-payment scenario, similar proportions of respondents preferred high intensity (higher duration/frequency, and face-to-face mode) and low intensity (lower duration/frequency, and remote mode) of the program. Although DCE evidence on such preference heterogeneity in lifestyle modification interventions remained limited, a systematic review showed that preferences for the frequency, duration, and intensity of exercises or counselling interventions were inconsistent across studies involving patients with different health needs [13]. These heterogeneous preferences suggest that offering a health coaching program with different intensity options may help promote program participation by meeting the expectations and needs of the target individuals (i.e., general middle-aged persons), who could have diverse values and preferences.

To our knowledge, this is one of the limited number of studies focusing on the preferences among middle-aged adults in the general population for a primary preventive, lifestyle promotion program. This study is also one of the few studies conducted among the Hong Kong population. The study therefore supplements the literature and provides a culturally specific perspective on how incentives for such programs can be designed. Most previous DCE studies on lifestyle interventions have focused on patients with established diseases or specific risk factors and have not primarily addressed prevention [17–19]. In addition, few studies have examined smartwatch provision as an attribute in a DCE setting. Our findings offer a distinct perspective on the potential role of smartwatch-based incentives in shaping preferences for health promotion programs.

This study has several limitations. First, the regression results for the overall study sample did not show a clear pattern of preferences for non-monetary attributes, including program duration, coaching intensity, and the format of different program components. This limited the ability of the study to identify a single optimal service delivery mode. Several factors may explain this finding. (1) Some respondents may have been inclined toward non-participation (as shown by the two-class latent class model in the sensitivity analysis). Therefore, the choices for the attribute levels may have been relatively random. (2) Respondents may not have had a clear preference for program design attribute levels after controlling for costs, but would prefer attribute levels associated with lower costs when copayment was incorporated into the preference weighting (as shown by the sensitivity analysis with copayment rate, rather than copayment amount, as an independent variable). (3) Respondents may have strong but heterogeneous, or even opposite, preferences for these attribute levels. Such heterogeneity may have been masked by the average preference estimates, resulting in average preference weights that were not statistically significantly different from zero, as shown by the four-class latent class model. A similar explanation of heterogeneous preferences was reported in a previous DCE study on a community-based care model [40]. This finding implies that public opinions and values regarding lifestyle interventions may vary, even though an introduction to the program was included before the DCE tasks in the questionnaire to standardise respondents’ understanding. Second, participants were recruited from a population-based cohort that evaluated long COVID outcomes. The sample may not fully represent the general Hong Kong population, which may limit the generalizability of the findings. In addition, females were overrepresented in the study sample (67.2%) compared with the general Hong Kong population (53.0%) [41]. This sex imbalance may further restrict the generalizability of the findings, as males may have different preferences compared with females [42]. While this is a common phenomenon, particularly in online surveys [43, 44], further studies are needed to target those with a lower education level to understand their perceptions and preferences for such a program. Third, a pilot survey to obtain priors for the preference weights of the attribute levels was not conducted because of resource constraints. Therefore, a zero-prior design, which has less efficiency, was adopted. Fourth, class 4 of the LCL model in the non-copayment scenario had a relatively small class share. While the number of classes was determined based on the goodness of fit of the overall latent class model, and therefore no single class should be omitted, the smaller class share may have resulted in relatively larger standard errors and greater uncertainty in the class-specific estimates. Fifth, 73 respondents consistently chose the opt-out option in the non-copayment scenarios, and 23 consistently chose the health coaching alternatives at the same position (either on the left- or right-hand side). Together, these respondents accounted for 11.1% of the 656 respondents. These response patterns suggest that some respondents may not have paid sufficient attention to the choice tasks, possibly due to the cognitive burden imposed by the tasks. These respondents were therefore excluded from the analysis, which may have reduced statistical power.

To advance Hong Kong’s primary healthcare reform, policymakers should prioritise affordability and digital integration as two key strategies to improve uptake of the preventive lifestyle program. Our results indicate that subsidising smartwatch provision and minimizing out-of-pocket expenses may be important for engaging middle-aged populations in lifestyle interventions. Integrating subsidised smartwatch provision, low copayment options, and remote coaching into the District Health Centre (DHC) framework through flexible, tiered coaching models that offer different levels of coaching intensity and delivery mode according to participants’ needs and preferences would enhance service accessibility. This strategy aligns with the Primary Healthcare Commission’s Co-care pilot and could contribute to a more prevention-oriented ecosystem that bridges the gap between clinical intervention and community-based self-management.

## Conclusions

In conclusion, this study used DCE to investigate the preferences of middle-aged adults for a lifestyle intervention program. The findings suggest that the copayment amount and the provision of a smartwatch were important factors associated with potential participants’ acceptance. Substantial preference heterogeneity was observed for non-monetary attributes across demographic groups. The findings may inform the design and delivery of health coaching programs for lifestyle modification, particularly within the local existing DHC framework. The results emphasise the importance of developing individualised interventions for lifestyle modification programs to meet diverse expectations of the general population. This suggests that offering flexible program options in program intensity, together with appropriate and carefully designed financial incentives, may help increase uptake among the middle-aged adults and thereby potentially improve population-level reach and effectiveness.

## Supplementary Information

Below is the link to the electronic supplementary material.

Supplementary Material 1

## Data Availability

The data supporting the findings of this study are not publicly available but may be obtained from the corresponding author upon reasonable request.
