# Supplementary material for: Individual preferences for the design of a health coaching-based lifestyle promotion program among middle-aged adults in Hong Kong: a discrete choice experiment
Source: Health Qual Life Outcomes. 2026 Jul 7;24:128. doi: 10.1186/s12955-026-02584-y (PMC13644059; doi:10.1186/s12955-026-02584-y)
Supplement: Supplementary file 1 [file 12955_2026_2584_MOESM1_ESM.docx]

**Supplementary Material**

**Supplementary Table A1. An example of effect coding for the attribute levels**

|  | X1 (6 months) | X2 (12 months) |
| --- | --- | --- |
| Program duration: 3 months (reference level) | -1 | -1 |
| Program duration: 6 months | 1 | 0 |
| Program duration: 12 months | 0 | 1 |

**Supplementary Table A2. Latent class logit model results in the “copayment” scenario**

|  | Class 1 | Class 2 | Class 3 | Class 4 |
| --- | --- | --- | --- | --- |
| Duration: 3 months | -0.29 (0.37) | -0.05 (0.06) | 0.11 (0.09) | 0.20 (0.11) |
| Duration: 6 months | 0.20 (0.31) | 0.02 (0.06) | 0.02 (0.07) | -0.11 (0.09) |
| Duration: 12 months | 0.09 (0.47) | 0.03 (0.08) | -0.13 (0.11) | -0.10 (0.13) |
| Coachingformat: Face-to-face | 0.46 (0.40) | 0.01 (0.07) | **-0.29 (0.12)** | **0.35 (0.09)** |
| Coachingformat: Videoconference | **-1.07 (0.50)** | 0.01 (0.05) | -0.07 (0.10) | -0.12 (0.09) |
| Coachingformat: WhatsApp/call | 0.61 (0.31) | -0.02 (0.07) | **0.36 (0.09)** | **-0.23 (0.09)** |
| Coachingfreq: every 2 weeks | -0.66 (0.45) | 0.01 (0.05) | 0.00 (0.09) | -0.09 (0.10) |
| Coachingfreq: every 3 weeks | 0.25 (0.31) | -0.01 (0.05) | 0.03 (0.08) | 0.00 (0.09) |
| Coachingfreq: every 4 weeks | 0.40 (0.36) | 0.00 (0.05) | -0.03 (0.10) | 0.09 (0.09) |
| Blood test: No such test | **0.76 (0.33)** | -0.08 (0.06) | -0.03 (0.10) | 0.04 (0.10) |
| Blood test: One test | **-1.19 (0.55)** | 0.04 (0.06) | 0.04 (0.08) | -0.15 (0.09) |
| Blood test: Two tests | 0.43 (0.41) | 0.04 (0.06) | -0.01 (0.10) | 0.11 (0.09) |
| Coreprogram: Face-to-face | -0.53 (0.36) | 0.05 (0.07) | -0.02 (0.11) | **0.27 (0.09)** |
| Coreprogram: Videoconference | -0.29 (0.36) | 0.03 (0.07) | -0.14 (0.08) | -0.16 (0.09) |
| Coreprogram: Videos | **0.82 (0.32)** | -0.08 (0.05) | 0.16 (0.10) | -0.10 (0.09) |
| Watch: Not necessary | 0.14 (0.30) | -0.05 (0.06) | -0.12 (0.09) | 0.03 (0.09) |
| Watch: One's own watch | -0.06 (0.30) | **-0.17 (0.05)** | **-0.35 (0.08)** | **-0.37 (0.10)** |
| Watch: Provided by program | -0.08 (0.37) | **0.22 (0.07)** | **0.47 (0.10)** | **0.34 (0.10)** |
| Out-of-pocket payment (every $100) | -0.03 (0.06) | 0.00 (0.01) | **-0.14 (0.02)** | **-0.08 (0.02)** |
| Opt-out | **5.10 (1.22)** | **-2.41 (0.25)** | -0.05 (0.06) | **-1.13 (0.30)** |
| Class share | 36.3% | 26.3% | 21.9% | 15.5% |
| Age: 45-59 years (vs 35-44 years) | REF | -0.11 (0.25) | -0.35 (0.27) | -0.37 (0.30) |
| Sex: female (vs male) | REF | **0.78 (0.26)** | **0.84 (0.28)** | **0.62 (0.31)** |
| Education: bachelor or higher (vs below bachelor) | REF | -0.20 (0.26) | 0.25 (0.28) | 0.22 (0.31) |
| Occupation: full time (vs others) | REF | -0.44 (0.27) | -0.18 (0.31) | -0.44 (0.32) |
| Obs | 16050 |  |  |  |
| N (participants) | 535 |  |  |  |
| LL | -3428.018 |  |  |  |
| BIC | 7543.562 |  |  |  |
| *Note: LL:log-likelihood ; BIC: Bayesian information criterion* | | | | |

**Supplementary Table A3. Latent class logit model results in the “no copayment” scenario**

|  | Class 1 | Class 2 | Class 3 | Class 4 |
| --- | --- | --- | --- | --- |
| Duration: 3 months | **-0.32 (0.05)** | **0.28 (0.06)** | -0.13 (0.18) | **0.31 (0.10)** |
| Duration: 6 months | **-0.12 (0.05)** | **0.12 (0.05)** | 0.16 (0.12) | -0.18 (0.10) |
| Duration: 12 months | **0.44 (0.07)** | **-0.40 (0.07)** | -0.03 (0.16) | -0.13 (0.10) |
| Coachingformat: Face-to-face | 0.11 (0.07) | **-0.41 (0.08)** | **0.44 (0.15)** | 0.11 (0.10) |
| Coachingformat: Videoconference | -0.05 (0.05) | **0.12 (0.05)** | **-0.73 (0.19)** | **-0.21 (0.11)** |
| Coachingformat: WhatsApp/call | -0.05 (0.05) | **0.28 (0.07)** | **0.29 (0.13)** | 0.10 (0.09) |
| Coachingfreq: every 2 weeks | 0.02 (0.05) | **-0.11 (0.05)** | **-0.64 (0.15)** | -0.12 (0.10) |
| Coachingfreq: every 3 weeks | 0.09 (0.05) | -0.04 (0.05) | -0.06 (0.13) | 0.11 (0.10) |
| Coachingfreq: every 4 weeks | **-0.11 (0.05)** | **0.15 (0.05)** | **0.70 (0.19)** | 0.01 (0.09) |
| Blood test: No such test | **-0.23 (0.06)** | 0.11 (0.06) | **0.56 (0.17)** | **0.34 (0.10)** |
| Blood test: One test | **0.16 (0.05)** | -0.10 (0.06) | -0.06 (0.13) | -0.11 (0.10) |
| Blood test: Two tests | 0.07 (0.05) | -0.01 (0.06) | **-0.50 (0.15)** | **-0.24 (0.10)** |
| Coreprogram: Face-to-face | **0.32 (0.06)** | **-0.36 (0.07)** | **-1.25 (0.26)** | 0.12 (0.11) |
| Coreprogram: Videoconference | **-0.11 (0.04)** | **0.16 (0.06)** | **0.66 (0.19)** | -0.13 (0.10) |
| Coreprogram: Videos | **-0.21 (0.05)** | **0.21 (0.05)** | **0.58 (0.16)** | 0.01 (0.10) |
| Watch: Not necessary | **-0.17 (0.05)** | **0.24 (0.06)** | **-1.54 (0.23)** | -0.01 (0.10) |
| Watch: One's own watch | **-0.17 (0.06)** | **-0.32 (0.06)** | **-1.94 (0.25)** | **-0.33 (0.11)** |
| Watch: Provided by program | **0.33 (0.07)** | 0.08 (0.07) | **3.48 (0.43)** | **0.34 (0.11)** |
| Opt-out | **-3.50 (0.35)** | **-2.77 (0.30)** | -0.57 (0.49) | -0.07 (0.26) |
| Class share | 38.6% | 34.2% | 15.5% | 11.7% |
| Age: 45-59 years (vs 35-44 years) | **0.76 (0.35)** | 0.45 (0.37) | 0.54 (0.41) | REF |
| Sex: female (vs male) | 0.61 (0.39) | 0.53 (0.41) | **1.31 (0.44)** | REF |
| Education: bachelor or higher (vs below bachelor) | -0.05 (0.35) | -0.31 (0.36) | -0.43 (0.41) | REF |
| Occupation: full time (vs others) | 0.39 (0.36) | 0.13 (0.37) | 0.32 (0.43) | REF |
| Obs | 16050 |  |  |  |
| N (participants) | 535 |  |  |  |
| LL | -4115.962 |  |  |  |
| BIC | 8880.716 |  |  |  |
| *Note: LL:log-likelihood ; BIC: Bayesian information criterion* | | | | |

**Supplementary Table A4. Sensitivity analysis on the effect of out-of-pocket payment presented in the choice set on participant choices in the “no copayment” scenario**

|  | Mean | SD |
| --- | --- | --- |
| Duration: 6 months | 0.01 (0.03) | -0.02 (0.06) |
| Duration: 12 months | -0.02 (0.04) | **0.44 (0.04)** |
| Coachingformat: Videoconference | -0.01 (0.03) | **-0.21 (0.05)** |
| Coachingformat: WhatsApp/call | **0.12 (0.03)** | **0.45 (0.04)** |
| Coachingfreq: every 3 weeks | 0.06 (0.03) | **0.11 (0.04)** |
| Coachingfreq: every 4 weeks | 0.02 (0.03) | 0.10 (0.05) |
| Blood test: One test | 0.00 (0.03) | **-0.23 (0.05)** |
| Blood test: Two tests | 0.01 (0.03) | 0.22 (0.06) |
| Coreprogram: Videoconference | 0.02 (0.03) | **0.26 (0.04)** |
| Coreprogram: Videos | 0.03 (0.03) | **-0.05 (0.08)** |
| Watch: One's own watch | **-0.42 (0.03)** | **0.15 (0.05)** |
| Watch: Provided by program | **0.50 (0.04)** | **0.26 (0.05)** |
| Out-of-pocket payment (every $100) | 0.00 (0.01) | **0.05 (0.00)** |
| Opt-out | **-3.08 (0.20)** | **2.43 (0.19)** |

**Supplementary Table A5. The mixed logit model with copayment rate as independent variables.**

|  | Mean (SE) | SD (SE) |
| --- | --- | --- |
| Duration: 3 months | **0.35 (0.04)** | 0.49 (0.05) |
| Duration: 6 months | 0.05 (0.03) | 0.05 (0.08) |
| Duration: 12 months | **-0.41 (0.05)** | 0.48 (0.05) |
| Coachingformat: Face-to-face | -0.07 (0.05) | 0.60 (0.05) |
| Coachingformat: Videoconference | -0.06 (0.04) | -0.23 (0.06) |
| Coachingformat: WhatsApp/call | **0.13 (0.04)** | 0.55 (0.05) |
| Coachingfreq: every 2 weeks | **-0.13 (0.04)** | 0.09 (0.06) |
| Coachingfreq: every 3 weeks | 0.02 (0.04) | 0.08 (0.06) |
| Coachingfreq: every 4 weeks | **0.11 (0.03)** | -0.05 (0.06) |
| Blood test: No such test | **0.10 (0.04)** | 0.16 (0.07) |
| Blood test: One test | 0.01 (0.04) | 0.01 (0.06) |
| Blood test: Two tests | **-0.10 (0.04)** | -0.16 (0.07) |
| Coreprogram: Face-to-face | 0.04 (0.04) | 0.18 (0.05) |
| Coreprogram: Videoconference | **-0.10 (0.04)** | 0.16 (0.05) |
| Coreprogram: Videos | 0.07 (0.04) | 0.08 (0.05) |
| Watch: Not necessary | **0.07 (0.04)** | 0.16 (0.06) |
| Watch: One's own watch | **-0.21 (0.04)** | 0.11 (0.06) |
| Watch: Provided by program | **0.14 (0.04)** | -0.12 (0.06) |
| Copayment rate: 100% | **-0.25 (0.04)** | 0.15 (0.05) |
| Copayment rate: 75% | **-0.08 (0.04)** | 0.06 (0.05) |
| Copayment rate: 50% | **0.33 (0.04)** | -0.13 (0.06) |
| Opt-out | 0.11 (0.20) | 6.46 (0.38) |

Note: Bolded figures refer to statistically significant coefficients (P<0.05).

**Supplementary Table A6. Results of the two-class models to segregate the non-participation.**

|  | Copayment | |  | Non-copayment | |
| --- | --- | --- | --- | --- | --- |
|  | Class 1 | Class 2 |  | Class 1 | Class 2 |
| Duration: 3 months | 0 | 0.21 (0.13) |  | 0 | **0.23 (0.08)** |
| Duration: 6 months | 0 | -0.12 (0.12) |  | 0 | -0.09 (0.08) |
| Duration: 12 months | 0 | -0.09 (0.17) |  | 0 | -0.14 (0.08) |
| Coachingformat: Face-to-face | 0 | **0.24 (0.11)** |  | 0 | 0.00 (0.08) |
| Coachingformat: Videoconference | 0 | **-0.27 (0.12)** |  | 0 | -0.10 (0.08) |
| Coachingformat: WhatsApp/call | 0 | 0.03 (0.11) |  | 0 | 0.10 (0.08) |
| Coachingfreq: every 2 weeks | 0 | -0.22 (0.14) |  | 0 | -0.13 (0.08) |
| Coachingfreq: every 3 weeks | 0 | 0.08 (0.11) |  | 0 | 0.11 (0.08) |
| Coachingfreq: every 4 weeks | 0 | 0.14 (0.11) |  | 0 | 0.02 (0.07) |
| Blood test: No such test | 0 | 0.00 (0.12) |  | 0 | **0.26 (0.08)** |
| Blood test: One test | 0 | -0.23 (0.12) |  | 0 | -0.08 (0.08) |
| Blood test: Two tests | 0 | 0.23 (0.12) |  | 0 | **-0.18 (0.08)** |
| Coreprogram: Face-to-face | 0 | 0.05 (0.13) |  | 0 | 0.04 (0.08) |
| Coreprogram: Videoconference | 0 | **-0.25 (0.12)** |  | 0 | -0.04 (0.08) |
| Coreprogram: Videos | 0 | 0.20 (0.11) |  | 0 | 0.00 (0.08) |
| Watch: Not necessary | 0 | -0.05 (0.11) |  | 0 | -0.07 (0.08) |
| Watch: One's own watch | 0 | **-0.37 (0.12)** |  | 0 | **-0.50 (0.09)** |
| Watch: Provided by program | 0 | **0.42 (0.13)** |  | 0 | **0.57 (0.08)** |
| Out-of-pocket payment (every $100) | 0 | **-0.07 (0.02)** |  | - | - |
| Opt-out | -1.81(0.08) | **2.01 (0.38)** |  | -3.80(0.20) | 0.08 (0.21) |

Note: Bolded figures refer to statistically significant coefficients (P<0.05).

**Supplementary Table A7. The mixed logit model results among the sample further excluding those with consistent choices at the same position in either one of the scenarios**

|  | "Copayment" scenario | |  | "No copayment" scenario | |
| --- | --- | --- | --- | --- | --- |
|  | Mean (SE) | SD (SE) |  | Mean (SE) | SD (SE) |
| Duration: 3 months | 0.05 (0.05) | **0.38 (0.06)** |  | -0.02 (0.04) | **0.52 (0.04)** |
| Duration: 6 months | 0.00 (0.04) | -0.10 (0.07) |  | 0.01 (0.03) | -0.10 (0.06) |
| Duration: 12 months | -0.04 (0.05) | **0.36 (0.06)** |  | 0.01 (0.04) | **0.51 (0.04)** |
| Coachingformat: Face-to-face | 0.01 (0.05) | **0.53 (0.05)** |  | **-0.11 (0.04)** | **0.53 (0.04)** |
| Coachingformat: Videoconference | -0.04 (0.04) | **0.29 (0.06)** |  | 0.01 (0.03) | **0.20 (0.06)** |
| Coachingformat: WhatsApp/call | 0.02 (0.04) | **0.44 (0.06)** |  | **0.09 (0.03)** | **0.49 (0.04)** |
| Coachingfreq: every 2 weeks | -0.02 (0.04) | 0.11 (0.06) |  | **-0.06 (0.03)** | 0.03 (0.06) |
| Coachingfreq: every 3 weeks | 0.02 (0.04) | 0.03 (0.06) |  | **0.08 (0.03)** | -0.01 (0.06) |
| Coachingfreq: every 4 weeks | 0.00 (0.04) | 0.10 (0.06) |  | -0.01 (0.03) | -0.03 (0.06) |
| Blood test: No such test | -0.03 (0.04) | **0.22 (0.07)** |  | -0.05 (0.03) | **0.26 (0.04)** |
| Blood test: One test | 0.01 (0.04) | **0.06 (0.06)** |  | 0.02 (0.03) | 0.03 (0.09) |
| Blood test: Two tests | 0.02 (0.04) | -0.21 (0.06) |  | 0.03 (0.03) | **0.25 (0.04)** |
| Coreprogram: Face-to-face | 0.06 (0.04) | **0.14 (0.06)** |  | 0.00 (0.03) | **0.25 (0.05)** |
| Coreprogram: Videoconference | -0.05 (0.04) | -0.05 (0.06) |  | 0.01 (0.03) | **0.13 (0.05)** |
| Coreprogram: Videos | -0.01 (0.04) | **0.13 (0.06)** |  | -0.01 (0.03) | **-0.21 (0.06)** |
| Watch: Not necessary | -0.03 (0.04) | **0.23 (0.07)** |  | **-0.07 (0.03)** | **0.22 (0.05)** |
| Watch: One's own watch | **-0.31 (0.04)** | -0.15 (0.08) |  | **-0.42 (0.03)** | 0.03 (0.06) |
| Watch: Provided by program | **0.34 (0.04)** | **0.17 (0.04)** |  | **0.49 (0.03)** | **0.21 (0.05)** |
| Out-of-pocket payment (every $100) | **-0.07 (0.01)** | **-0.07 (0.01)** |  | - | - |
| Opt-out | **-1.34 (0.21)** | **5.43 (0.27)** |  | **-3.55 (0.24)** | **2.82 (0.17)** |

Note: Bolded figures refer to statistically significant coefficients (P<0.05).

**Supplementary Figure A1. Relative importance percentage of the attributes of the two scenarios in mixed logit model**

|  | Copayment | Non-copayment |
| --- | --- | --- |
| Duration | 8.33404% | 3.68402% |
| Coaching format | 3.30741% | 14.2618% |
| Coaching frequency | 2.04655% | 9.73572% |
| Blood test | 3.22472% | 3.71588% |
| Core program format | 9.18864% | 3.68836% |
| Provision of watch | 37.9022% | 64.9142% |
| Payment (HK$0-1000) | 35.9965% |  |
